# Prognostic value of KRAS subtype in patients with PDAC undergoing radical resection

**DOI:** 10.3389/fonc.2022.1074538

**Published:** 2022-12-13

**Authors:** Manxiong Dai, Raja Jahanzaib, Yan Liao, Fengxuan Yao, Jia Li, Xiong Teng, Kang Chen, Wei Cheng

**Affiliations:** ^1^ Department of Hepatobiliary Surgery, Hunan Provincial People’s Hospital, The First Affiliated Hospital of Hunan Normal University, Changsha, Hunan, China; ^2^ Translational Medicine Laboratory of Pancreas Disease of Hunan Normal University, Changsha, China; ^3^ Xiangyue Hospital Affiliated to Hunan Institute of Parasitic Diseases, National Clinical Center for Schistosomiasis Treatment, Yueyang, Hunan, China

**Keywords:** pancreatic ductal adenocarcinoma, genomic mutation, next-generation sequencing, KRAS, prognosis, radical surgery

## Abstract

**Objective:**

To explore the frequency distribution of KRAS mutant subtypes in patients with resectable PDAC in China and then evaluate the prognostic value of different KRAS subtypes in patients with PDAC undergoing radical resection.

**Methods:**

The clinicopathological data and gene test reports of 227 patients undergoing PDAC radical surgery at Hunan Provincial People’s Hospital from 1 January 2016 to 1 January 1 2020 were retrospectively evaluated. There were 118 men (52%) and 109 women (48%). The mean age was 58.8 ± 10.3 years. After univariate analysis of the clinicopathological factors (sex, age, presence or absence of underlying disease, location of the primary tumour, tumour TNM stage, T stage, N stage, presence or absence of vascular invasion, presence or absence of nerve invasion, surgical margin, KRAS mutation subtype), variables with P < 0.1 were included in the multivariate Cox regression model analysis, and the log-rank sum test and Kaplan−Meier curves were used to assess the correlation of the KRAS mutation subtype with the overall survival time.

**Results:**

KRAS mutations were detected in 184 of 227 patients (81.1%) (G12D: 66; G12V: 65; G12R: 27; Q61:26) and were not detected in 43 patients (18.9%). KRAS mutations were associated with tumour differentiation (P = 0.001), TNM stage (P = 0.013), and T stage (P < 0.001). Multivariate Cox regression model analysis showed that N stage, surgical margin, tumour differentiation, and KRAS-G12D mutation were independent prognostic factors for DFS and OS. Patients with the KRAS-G12D subtype had shorter OS with a median OS of 12 months (HR: 0.55, CI: 0.39–0.77, P < 0.001), and patients with KRAS wild-type had longer OS with a median OS of 19 months (HR: 0.57, CI: 0.42–0.76, P < 0.001).

**Conclusion:**

KRAS wild-type individuals are more prevalent in the Chinese population than in European or American populations. Patients undergoing surgery had a reduced percentage of tumors with KRAS-G12D. When determining the prognosis of individuals with radically resected PDAC, reference markers for KRAS mutation subtypes can be employed.

## Introduction

Pancreatic ductal adenocarcinoma (PDAC) is one of the most malignant tumours, with a 5-year survival rate of less than 10% (1). Surgery is the only means of achieving a radical cure, but less than 15% of patients have the chance of surgery when diagnosed with PDAC. Furthermore, the vast majority of operable patients will relapse within 1–2 years after surgery (2, 3). From the first open pancreaticoduodenectomy (OPD) in 1898 to the first total laparoscopic pancreaticoduodenectomy (LPD) in 1994 (4) and, more recently, the development of robotic surgery, surgical techniques and tools for PDAC resection have developed rapidly. However, the 5-year survival rate of patients is only slowly increasing, which is closely related to the heterogeneity and drug resistance of pancreatic tumours as well as their unique tumour microenvironment (5, 6). The development of PDAC treatment in the future will primarily focus on precision treatment based on next-generation gene sequencing technology (NGS). A key factor in improving PDAC patient survival time is finding more target genes and pathogenic pathways to accurately stratify patients so they can receive the most optimal treatment.

In 2008, Jones et al. (7) first determined the whole exon sequence of PDAC and revealed that it had different gene mutations in all 12 pathways of tumour formation. An increasing number of studies have found that PDAC has four high-frequency gene mutations (KRAS, TP53, SMAD4, CDKN2A), among which KRAS mutations are found in 90% of PDAC patients, and this proportion is the highest of all solid tumours (8). KRAS is a proto-oncogene that plays an important role in the regulation of life activities such as cell growth and angiogenesis. Once mutated, it leads to the continuous growth of cells and ultimately to the development of PDAC (9, 10). KRAS mutations occur at codon 12 in 95% to 97% of PDAC patients. Missense mutations occur most frequently and can be divided into G12D, G12V, G12R, G12C, G12A, and G12S according to the base of the missense mutation, and other less frequent mutations can also occur at codons 13, 61, 117, and 146 (11). Some previous studies have found that KRAS-G12D mutation is an independent risk factor for a poor prognosis of PDAC. KRAS wild-type patients have longer overall survival time (OS) and disease-free survival (DFS) (12–16). However, no large sample size studies have performed survival analysis on patients with resectable pancreatic cancer with different KRAS subtypes. The vast majority of the research is conducted on patients with advanced PDAC, utilizing specimens of metastases to the liver, and integrating clinicopathologic and surgical specimen sequencing data. We investigated the prevalence of KRAS subtypes in resectable PDAC patients in China and evaluated the predictive significance of KRAS subtypes in PDAC patients who received radical surgical resection.

## Methods

### General data

We retrospectively investigated the clinicopathological data and genetic testing reports of 227 patients who underwent PDAC radical surgery (including laparotomy, endoscopic and robotic pancreaticoduodenectomy and distal pancreatectomy) in Hunan Provincial People’s Hospital between 1 January 2016 and 1 January 2020. Underlying diseases included diabetes mellitus, hypertension, coronary heart disease, asthma and stroke. Pathological TNM staging was performed according to the American Joint Committee on Cancer (AJCC) 8th edition classification of tumour-node-metastasis of pancreatic cancer. Negative surgical margins (R0) were defined as cancer cells having a distance greater than 1 mm from the resection margin. This study was approved by the medical ethics committee of our hospital, and the batch number was (49). Patients and their families signed informed consent forms.

Inclusion criteria: ①Pathological diagnosis of PDAC; ②Radical resection of PDAC and gene sequencing of the surgical specimens; and ③Signed the consent form.

Exclusion criteria: ①Patients who died within 90 days after surgery; ②Patients who received neoadjuvant chemotherapy before surgery; ③Patients who did not receive adjuvant chemotherapy after surgery. Postoperative adjuvant chemotherapy regimens included oral S-1 alone, the AG chemotherapy regimen, the FOLFIRINOX chemotherapy regimen, and the gemcitabine monotherapy chemotherapy regimen; and ④ Lack of follow-up data due to loss of contact.

### Postoperative follow-up and adjuvant chemotherapy

Outpatient and inpatient reexamination follow-up or telephone follow-up was performed every 3 months within 2 years after surgery and every 6 months within 2-5 years after surgery. Tumour recurrence, time to tumour recurrence, death, cause of death, and time to death were recorded. OS and DFS were calculated until 01 September 2022. If the patient did not die during the follow-up, the survival time was determined as the last follow-up time. Adjuvant chemotherapy was administered 1 month after surgery. The choice of postoperative adjuvant chemotherapy regimen was based on the patient’s compliance and physical condition, and included ① oral S-1, 80 mg – 120 mg/day, continuous oral administration for 21 days, 14 days off, continued for 6 months; ② intravenous albumin paclitaxel + gemcitabine (AG), injection on Days 1 and 8, 3 weeks per cycle. The albumin paclitaxel dose was 125 mg/m^2^, the gemcitabine dose was 1000 mg/m^2^, and this regimen continued until 4–6 months after surgery; ③ intravenous gemcitabine alone, injection on Days 1 and 8, 3 weeks per cycle. The gemcitabine dose was 1000 mg/m^2^, and it continued until 4–6 months after surgery; ④ FOLFIRINOX chemotherapy regimen: intravenous oxaliplatin 85 mg/m^2^, irinotecan 180 mg/m^2^, leucovorin 400 mg/m^2^, and fluorouracil 400 mg/m^2^, followed by continuous intravenous infusion of fluorouracil 2400 mg/m^2^, and continuous intravenous infusion of 400 mg/m^2^ on the first day of each course of treatment. This regimen was repeated every 2 weeks for 4-6 months after surgery.

### Acquisition of NGS sequencing data

Prior to sample collection, the patient’s basic information was recorded. The tumour tissue was obtained from the resected specimen within 5 minutes after surgery and was placed in a cooler at 0~4°C and then sent to the laboratory within 2 hours. After arriving at the laboratory, only if the tumour tissue was pathologically confirmed and the tumour cell content was greater than 20% was the tumour tissue subjected to DNA extraction. The following criteria were adopted for DNA quality control: the concentration was greater than 50 g/L, the A260/A280 value was between 1.8 and 1.9, and the DNA appeared as a clear and brilliant single band after electrophoresis on a 1% agarose gel. Next, sequencing libraries were constructed using the Ion AmpliSeq Kit for Chef DL8 (A29024, Thermo Scientific) in conjunction with the Ion Comprehensive Cancer Panel Primer Pool (4477685, Thermo Scientific). Templates were prepared on the Ion Chef using the Ion P1 Hi-Q Chef Kit (A27198, Thermo Scientific). A P1 Chip v3 was used with an Ion Proton sequencer to sequence the templates to a minimum of 500X mean coverage (A26771, Thermo Scientific). The raw sequenced data were then processed for quality control, and the low-quality reads were excluded. For somatic variant and copy number calling, genomic data (BAM files) were imported into Ion Reporter (Thermo Scientific). Additionally, second-level annotation was performed using Ingenuity Variant Analysis (Qiagen).

### Statistical analysis

SPSS 26.0 statistical software was used for data analysis. Enumeration data are expressed as the number of cases (percentage), measurement data are expressed as the mean ± standard deviation (x ± SD), and OS and DFS are expressed as the median. Comparisons between groups were accomplished using the chi-square test or Fisher’s exact test. To identify factors that may influence the prognosis, a Cox proportional hazards regression model was established for application in univariate and multivariate survival analyses. Survival analysis was performed using the Kaplan−Meier method and the log-rank sum test. To be statistically significant, P < 0.05.

## Result

### Clinical data of 227 patients

Among 227 PDAC patients, 118 (52%) were men and 109 (48%) were women; their mean age was 58.8 ± 10.3 years; 194 (85.5%) had primary tumours located in the head of the pancreas and 33 (14.5%) had primary tumours located in the body and tail of the pancreas; and 10 patients (4.4%) had R1 resection margins. A total of 217 patients (95.6%) had R0 margins (**Table 1**).

**Table 1:** Main characteristics of the patients.

Characteristic	Total	KRAS-G12D	KRAS-G12V	KRAS-G12R	KRAS-Q61	Wild-type	P-Value
Gender							0.745
male	118	36	37	12	12	21	
female	109	30	28	15	14	22	
Age							0.562
≥65	70	22	24	7	7	10	
< 65	157	44	41	20	19	33	
Basic disease							0.142
no	139	46	33	14	17	29	
yes	88	20	32	13	9	14	
TNM stage							0.013
I	126	34	32	21	9	30	
II	80	25	27	6	11	11	
III	21	7	6	0	6	2	
T stage							<0.001
T1	23	6	3	2	0	12	
T2	152	46	40	25	15	26	
T3	52	14	22	0	11	5	
N stage							0.056
N0	157	42	48	21	12	34	
N1	49	17	11	6	8	7	
N2	21	7	6	0	6	2	
Tumor location							0.698
head	194	58	57	23	20	36	
body/tail	33	8	8	4	6	7	
Differentiation							0.001
poorly	118	47	25	12	15	19	
moderately	88	12	34	14	11	17	
well	21	7	6	1	0	7	
Vascular invasion							0.993
no	197	57	56	23	23	38	
yes	30	9	9	4	3	5	
Perineural invasion							0.816
no	16	4	4	3	1	4	
yes	211	62	61	24	25	39	
Surgical margin							0.760
R0	217	64	61	26	24	42	
R1	10	2	4	1	2	1	

### Sequencing data

**Figure 1:**
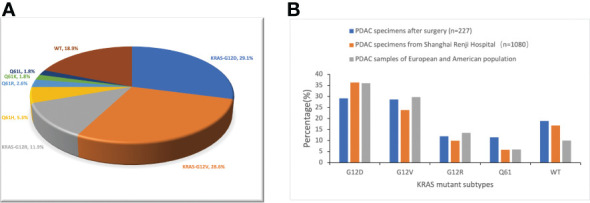
Frequency distribution of KRAS mutation subtypes in pancreatic cancer patients. **(A)** Distribution of KRAS mutations in 227 PDAC surgical samples from Hunan Provincial People’s Hospital. WT, KRAS wild type. **(B)** Differences in the frequency distribution of KRAS subtypes. Blue represents the data of our study. Not only surgical samples, but also needle biopsy samples of advanced PDAC, are included in the analysis of KRAS subtype distribution in the Shanghai Renji Hospital, European, and American populations.

According to the reports of postoperative gene sequencing, 66 patients (29.1%) had KRAS-G12D mutation, 65 patients (28.6%) had KRAS-G12V mutation, 27 patients (11.9%) had KRAS-G12R mutation, 12 patients (5.3%) had KRAS-Q61H mutation, 6 patients (2.6%) had KRAS-Q61R mutation, 4 patients (1.8%) had KRAS-Q61K mutation, and 4 patients (1.8%) had KRAS-Q61L mutation. In addition, 43 patients (18.9%) were identified as “KRAS wild-type”, signifying that no mutation in the KRAS gene was discovered. These 227 patients were split into five groups according to the distribution of their KRAS mutations: KRAS-G12D (29.1%), KRAS-G12V (28.6%), KRAS-G12R (11.9%), KRAS-Q61 (11.5%), and KRAS wild-type (18.9%) (**Figure 1A**). In patients with detected KRAS gene mutations, all were single base point mutations, and two or more base mutations or other forms of mutation were not detected. We compared our data with the data from Shanghai Renji Hospital (17) and European and American populations (18) (**Figure 1B**).

The results of the correlation analyses between the five KRAS mutation subgroups and the clinicopathological data of the patients suggested that KRAS mutation was associated with T stage (P < 0.001). Similarly, KRAS mutation was significantly associated with tumour differentiation (P < 0.001). There was no substantial difference among the five groups with respect to sex, age, presence or absence of underlying disease, location of the main tumour, TNM stage of the tumour, N stage, presence or absence of vascular invasion, presence or absence of nerve invasion, or surgical margin (P > 0.05) (**Table 1**).

### Univariate and multivariate analysis of prognostic factors in patients after PDAC

Univariate analysis of factors correlated with OS and DFS in PDAC patients showed that age, TNM stage, T stage, N stage, tumour differentiation, resection margin, presence of KRAS mutation, and presence of KRAS wild-type were associated with OS of PDAC patients (P < 0.05) (**Table 2**). Age, TNM stage, T stage, N stage, tumour differentiation, resection margin, presence of KRAS mutation, and KRAS wild-type status were associated with DFS after resection (P < 0.05) (**Table 3**). However, other KRAS subtypes, including KRAS-G12V, KRAS-G12R, and KRAS-Q61 mutations, were not associated with OS or DFS.

**Table 2:** Univariate analysis of factors affecting patients ‘overall survival in pancreatic ductal adenocarcinoma.

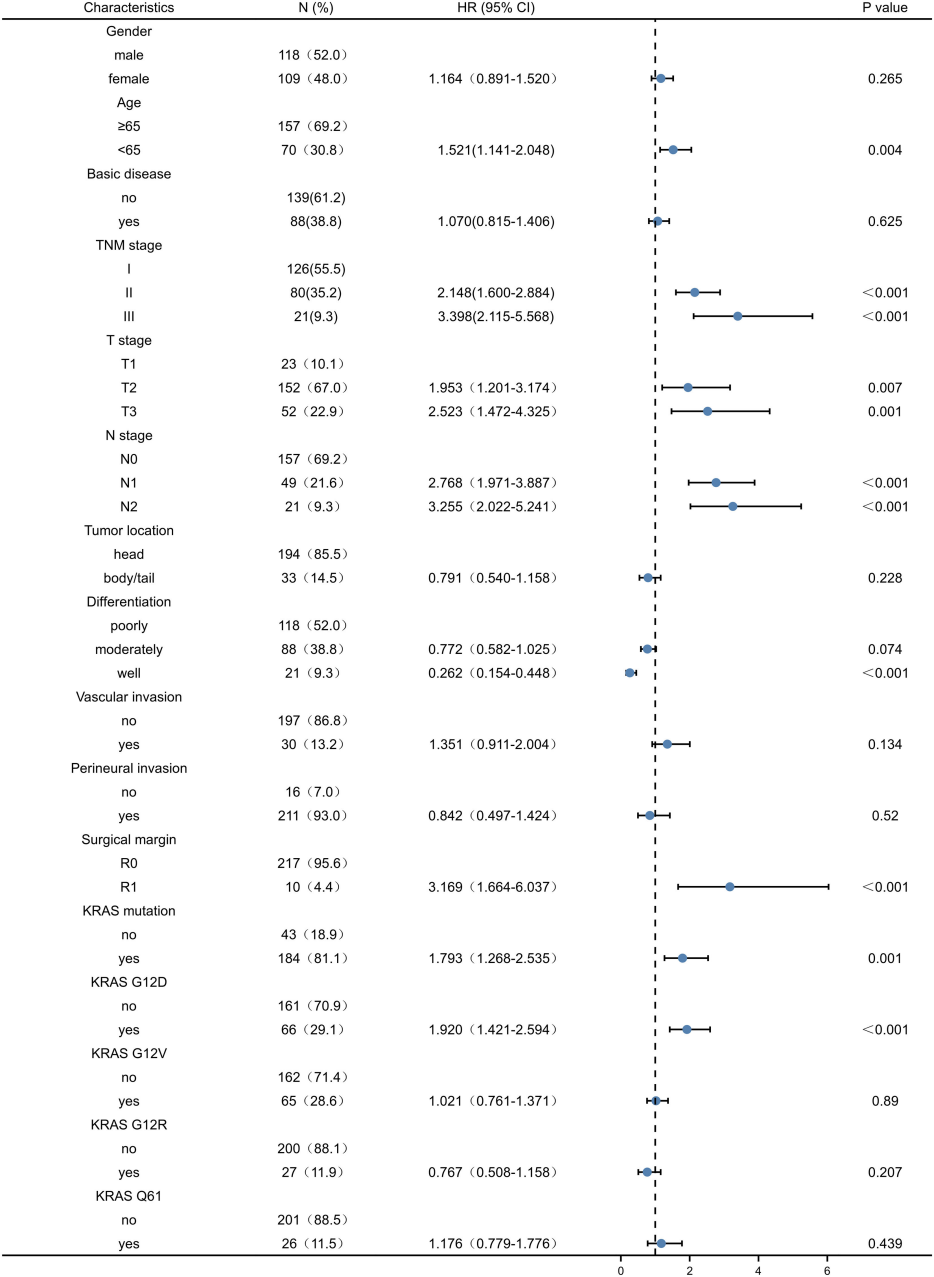

95%CI, 95% confidence interval; HR, hazard ratio. The blue dot is HR, and the interval of the line is 95%CI.

**Table 3:** Univariate analysis of factors affecting patients’ disease-free survival in pancreatic ductal adenocarcinoma.

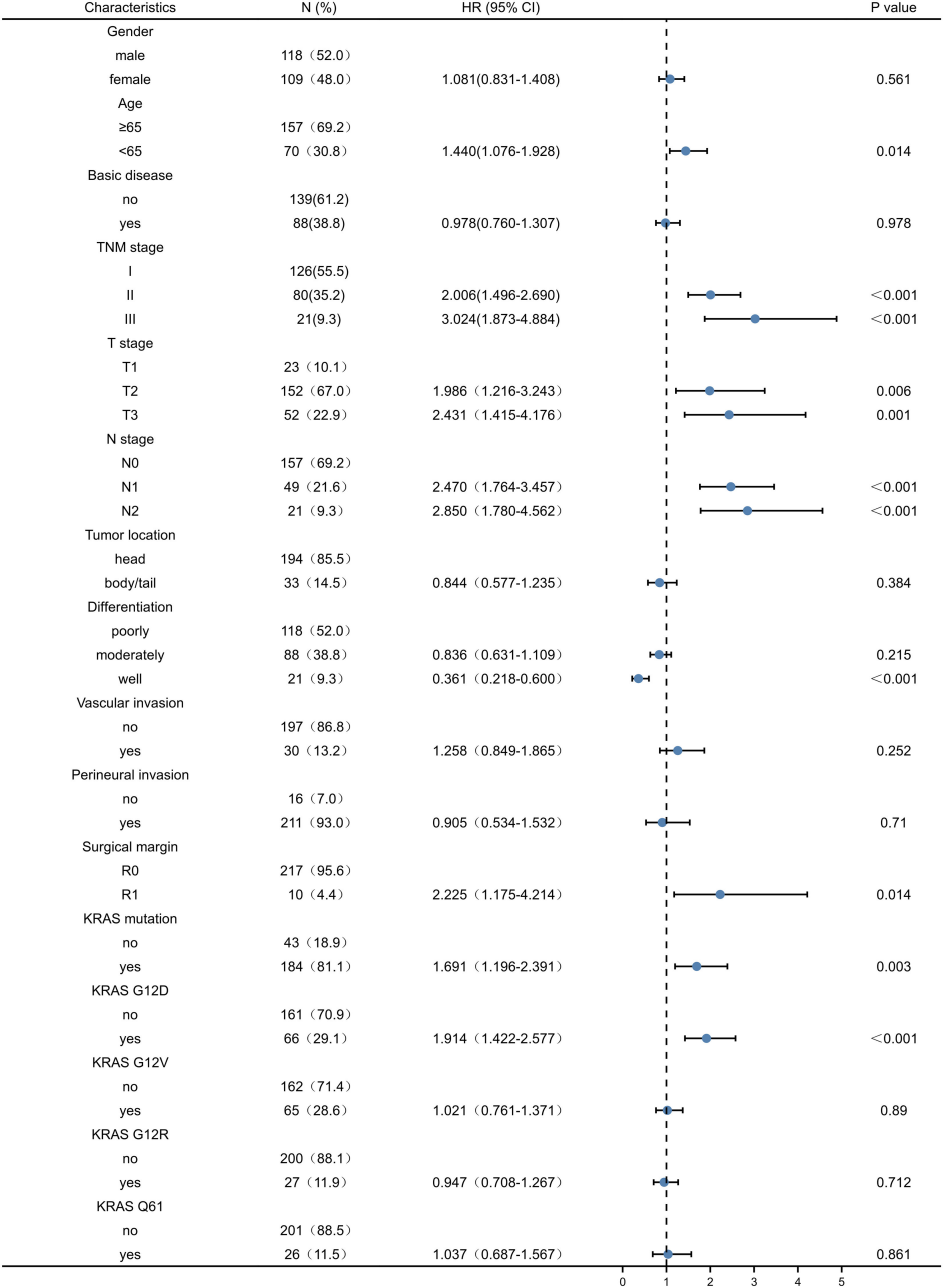

95%CI, 95% confidence interval; HR, hazard ratio. The blue dot is HR, and the interval of the line is 95%CI.

Including the variables with P < 0.1 in the above univariate analyses in the multivariate Cox regression model, we found that TNM stage III, N stage, surgical margin, well-differentiated tumours, and KRAS-G12D mutation were independent prognostic factors for OS. N stage, well-differentiated tumour, surgical margin, and KRAS-G12D mutation were independent prognostic factors for DFS (**Table 4**).

**Table 4:** Multivariate analysis of factors affecting patients’ OS and DFS in pancreatic ductal adenocarcinoma.

characteristics	N (%)	OS		DFS	
		HR (95%CI)	P value	HR (95%CI)	P value
Age					
<65	157 (69.2)				
≥65	70 (30.8)	1.411 (1.036-1.922)	0.029	1.270 (0.929-1.737)	0.134
TNM stage					
I	126 (55.5)				
II	80 (35.2)	1.144 (0.606-2.160)	0.678	1.183 (0.617-2.266)	0.613
III	21 (9.3)	2.975 (1.663-5.324)	<0.001	1.291 (0.503-3.313)	0.596
T stage					
T1	23 (10.1)				
T2	152 (67.0)	1.560 (0.923-2.634)	0.097	1.509 (0.887-2.567)	0.129
T3	52 (22.9)	1.603 (0.792-3.242)	0.190	1.476 (0.715-3.047)	0.293
N stage					
N0	157 (69.2)				
N1	49 (21.6)	2.649 (1.454-4.823)	<0.001	2.456 (1.678-3.556)	<0.001
N2	21 (9.3)	2.667 (1.478-4.904)	<0.001	2.509 (1.780-3.786)	<0.001
Differentiation					
poorly	118 (52.0)				
moderately	88 (38.8)	0.871 (0.650-1.167)	0.355	0.950 (0.706-1.278)	0.735
well	21 (9.3)	0.251 (0.145-0.437)	<0.001	0.370 (0.219-0.624)	<0.001
Surgical margin					
R0	217 (95.6)				
R1	10 (4.4)	4.396 (2.173-8.895	<0.001	2.934 (1.428-6.028)	<0.001
KRAS mutation					
no	43 (18.9)				
yes	184 (81.1)	1.136 (0.770-1.676)	0.521	1.024 (0.690-1.519)	0.906
KRAS G12D					
no	161 (70.9)				
yes	66 (29.1)	2.261 (1.614-3.169)	<0.001	2.173 (1.553-3.041)	<0.001

### Relationship between different KRAS subgroups and the prognosis of patients after PDAC

**Figure 2:**
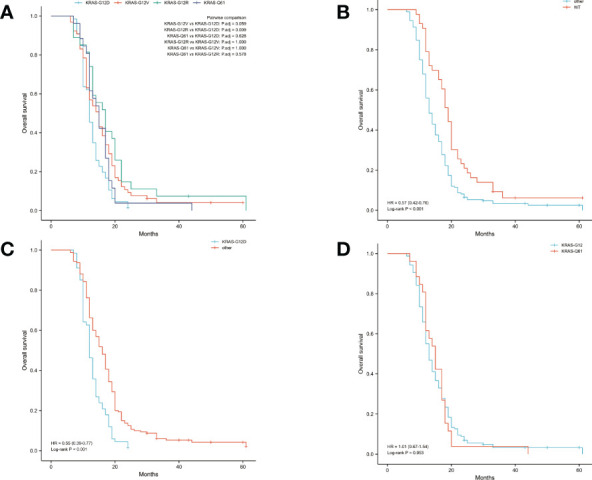
Overall survival (OS) in all patients was examined with Kaplan-Meier plots and log-rank tests. **(A)** Comparison of four groups (KRAS-G12D, KRAS-G12V, KRAS-G12R, KRAS-Q61) with KRAS mutations. **(B)** Comparison of patients with KRAS wild-type and those with KRAS mutations. Other groups included KRAS-G12D, KRAS-G12V, KRAS-G12R, and KRAS-Q61. **(C)** Intercomparison between patients with KRAS-G12D mutation and other patients. Other groups included KRAS wild-type, KRAS-G12V, KRAS-G12R, and KRAS-Q61. **(D)** Examination of mutations at codons 12 and 61. KRAS-G12D, KRAS-G12V, and KRAS-G12R were all part of the codon 12 group of KRAS. The KRAS-Q61 family consisted of four different variants (KRAS-Q61H, KRAS-Q61R, KRAS-Q61K, and KRAS-Q61L). WT, KRAS wild-type; HR, hazard ratio; The numbers in parentheses represent 95% confidence intervals.

**Figure 3:**
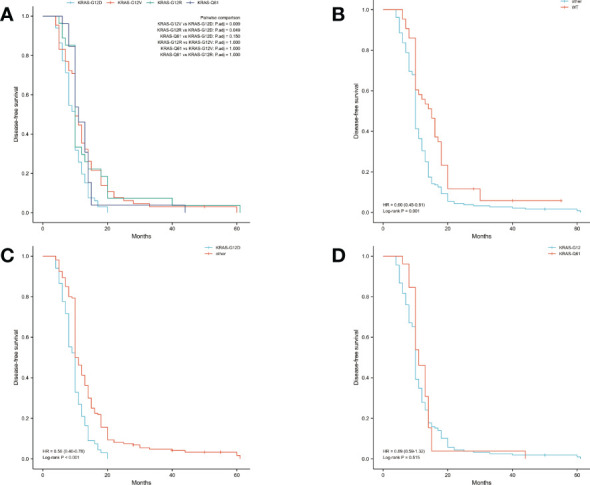
Disease-free survival (DFS) in all patients was examined with Kaplan-Meier plots and log-rank tests. **(A)** Comparison of four groups (KRAS-G12D, KRAS-G12V, KRAS-G12R, KRAS-Q61) with KRAS mutations. **(B)** Evaluation of KRAS mutation carriers against individuals with the wild-type gene. Additional subsets were denoted by the names KRAS-G12D, KRAS-G12V, KRAS-G12R, and KRAS-Q61. **(C)** Those with the KRAS-G12D mutation were compared to those without it and vice versa. Wild-type KRAS, KRAS-G12V, KRAS-G12R, and KRAS-Q61 were the other subsets. **(D)** A comparison of the mutations at codons 12 and 61. The KRAS-G12D, V, and R codon 12 groups were present. The KRAS-Q61H, Q61R, Q61K, and Q61L codons were part of the codon 61 group. WT, KRAS wild-type; HR, hazard ratio; The numbers in parentheses represent 95% confidence intervals.

The median OS was 14 months and the median DFS was 10 months for all 227 patients. The median OS was shortest in the KRAS-G12D group, only 12 months, while the median DFS was 10 months. The median OS and median DFS was, respectively, 15 and 10 months in the KRAS-G12V group; 17 and 10 months in the KRAS-G12R group; 15 and 11 months in the KRAS-Q61 group; and 19 and 15 months in the KRAS wild-type group (**Figures 2**, **3**).

We performed pairwise comparisons between the 4 groups with KRAS mutations (KRAS-G12D, KRAS-G12V, KRAS-G12R, KRAS-Q61), and patients in the KRAS-G12D group had shorter OS and DFS than patients in the KRAS-G12V group and patients in the KRAS-G12R group (P < 0.05), but there was no statistically significant difference in OS and DFS compared with patients in the KRAS-Q61 group (P = 0.628, P = 0.150). There was no significant difference in OS and DFS among the KRAS-G12V, KRAS-G12R, and KRAS-Q61 groups (**Figure 2A**, **Figure 3A**). We divided the 227 patients into a KRAS mutation group (including KRAS-G12D, KRAS-G12V, KRAS-G12R, KRAS-Q61 groups) and a KRAS-wild-type group according to the presence or absence of KRAS mutation and compared the OS and DFS between these two groups. We found that patients in the KRAS-wild-type group had longer OS and DFS (HR: 0.57, CI: 0.42–0.76, P < 0.001; HR: 0.60, CI: 0.45–0.81, P = 0.001) (**Figure 2B**, **Figure 3B**). We divided the 227 patients into the KRAS-G12D group and the other patient groups (including KRAS-G12V, KRAS-G12R, KRAS-Q61, and KRAS-wild type), compared the OS and DFS between these two groups, and found that the KRAS-G12D group had shorter OS and DFS (HR: 0.55, CI: 0.39–0.77, P < 0.001; HR: 0.56, CI: 0.40–0.78, P < 0.001) (**Figure 2C**, **Figure 3C**). Then, KRAS mutations located in codon 12 were grouped and those in codon 61 were grouped, and the OS and DFS were compared between these two groups, but there was no significant difference in OS and DFS (HR: 1.01, CI: 0.67–1.54, P = 0.953; HR: 0.89, CI: 0.59–1.32, P = 0.515) (**Figure 2D**, **Figure 3D**).

## Discussion

The KRAS protein is a nearly spherical structure with no obvious binding sites, and it is difficult to synthesize a compound that can target it and inhibit its activity (19, 20). In 2013, Shoket and his team (21) discovered an allosteric binding pocket behind switch-II of the KRAS-G12C protein, a finding that spawned several small molecule covalent inhibitors targeting the KRAS G12C mutation. Among them, Amgen’s sotorasib (AMG510) was approved by the FDA in May 2021 for patients with KRAS G12C-mutated non-small cell lung cancer and is the first KRAS-targeted drug marketed worldwide. AMG510 showed promising results in phase I and II clinical trials for advanced solid tumours, including 8 stable disease (SD) and 1 partial response (PR) in 12 enrolled patients with advanced PDAC (22). With 90% of PDAC patients carrying KRAS mutations, the therapeutic potential of KRAS-targeted drugs is vast (23). Although the frequency of the KRAS-G12C mutation is less than 1% (24), the marketing of AMG-510 has opened a new era of directly targeting KRAS, and targeted drugs against other subtypes, such as KRAS-G12D, are also being developed. With the advancement of NGS technology, an increasing number of PDAC patients are seeking NGS technology with the goal of developing individualized treatment plans. There have been thousands of completed or ongoing clinical trials for PDAC patients. However, as of today, no targeted drug for PDAC has been approved for marketing. This reflects the complexity of precision treatment for PDAC, such as determining how to correctly use gene sequencing data to match individual PDAC patients with the most optimal customized treatment options. This a challenging task for clinicians.

Contrasting our data on KRAS mutations with findings in Western populations of PDAC patients, we found (11, 18, 25, 26) that more Chinese had undetectable KRAS mutations and were classified as KRAS wild-type. Additionally, we discovered a reduced percentage of patients with KRAS-G12R mutations. These variations and modifications are similar to the PDAC gene mutation profiles in Chinese populations that were previously published (17, 27). Fascinatingly, patients with wild-type KRAS frequently have mutations in other genes, including BRAF, EGFR, MET, and KIT (28). In this subset of patients, the EGFR-targeted inhibitor nimotuzumab combined with gemcitabine significantly improved OS in patients with advanced PDAC by 6 months compared with gemcitabine alone (29). In view of the high proportion of KRAS wild-type Chinese PDAC patients, we have more patients who can benefit from EGFR-targeted inhibitors such as nimotuzumab. Relapsed or progressing KRAS wild-type patients treated with first-line adjuvant chemotherapy can benefit from the addition of targeted inhibitors of other targets, such as EGFR. No mutations were found in codon 13 or the recently discovered hotspot KRAS-G12C in our sample of 227 patients. Despite the fact that we did not find a correlation between KRAS mutations and age, earlier research indicated that KRAS mutations are more prevalent in elderly patients (17, 30). Lu et al. (31) found that KRAS mutations were more common in tumours in the body and tail of the pancreas than in tumours in the head and neck of the pancreas. Our study did not find similar results, which may be related to the differences in the study population. Lu et al. studied patients with pancreatic cancer at various stages, while we focused on patients with early resectable pancreatic cancer. Compared with the data of 1080 patients with PDAC (including surgical samples and needle biopsy samples from patients with advanced PDAC) from Shanghai Renji Hospital, it was found that the detection rate of KRAS-G12D in our PDAC surgical samples was lower. This may be due to the fact that KRAS-G12D patients have a higher TNM stage and more patients lose the chance of surgery when pancreatic cancer is diagnosed, resulting in a decrease in the proportion of KRAS-G12D patients among surgical patients (18).

In this study, we discovered that the N stage of the tumour and the R1 resection margin were independent risk factors for poor outcomes in patients with PDAC for both OS and DFS. As a consequence, it was crucial to perform an intraoperative rapidly frozen biopsy away from the tumour to correctly transect and section the pancreas. Patients in the KRAS-G12D subgroup exhibited shorter OS and DFS than patients in the other KRAS mutant subgroups, whereas patients with wild-type KRAS had longer OS and DFS, and comparable findings have been reported in prior investigations (13–16). However, most of the subjects of these prior studies were patients with advanced PDAC, and most of the specimen sources were needle biopsies, with a small sample size. Some studies have demonstrated that PDAC specimens are heterogeneous, with large differences in gene mutation types between primary tumours and metastases and some differences in differentiation as well as growth rates (32). A large proportion of these studies in patients with advanced PDAC were conducted on metastases, such as to the liver, so this also explains why the key data in these studies, such as the rates of KRAS and CDKN2A gene mutations, are quite different from large-scale gene sequencing data obtained in recent years. Our study subjects were mainly patients with resectable early PDAC. The samples were obtained from the primary tumour after the operation. After multipoint sampling and pathological confirmation, the sample quality and tumour cell content were superior to those obtained in previous studies *via* endoscopic or ultrasound-guided fine-needle aspiration biopsy, which could minimize the impact of tumour heterogeneity on the sequencing results. Through these results, we can predict the outcome of patients according to their postoperative gene sequencing report, communicate the severity of the disease to the patients and their families, and develop a more individualized follow-up plan. For patients with the KRAS-G12D subtype of postoperative pancreatic cancer, more frequent follow-up times and more comprehensive and imaging examinations are necessary. We grouped all mutations located in KRAS codon 61 together, and there was no significant difference in OS and DFS compared with patients with mutations located in KRAS codon 12, indicating that the codon in which KRAS mutations are located has little relationship with the prognosis of the patients.

This study has several limitations: ① A single-centre retrospective investigation was conducted. ② Due to the limitations of the sample size, survival analysis of KRAS-Q61H, KRAS-Q61R, KRAS-Q61K and KRAS-Q61L was not performed separately for the analysis of KRAS-Q61 subtypes, and survival analysis of these low-frequency mutations needs to be conducted in larger studies. ③ The influencing factor of postoperative adjuvant chemotherapy was not considered separately. Because the postoperative adjuvant chemotherapy regimens basically included the two most commonly used first-line treatment regimens (AG, FOLFIRINOX), and patients tended to switch from one chemotherapy regimen to the other chemotherapy regimen if they progressed, it was challenging to use the chemotherapy regimen to group patients. It is essential to investigate how patients with distinct KRAS subtypes respond to different chemotherapy regimens. ④ Pancreatic tumours have adhesion hyperplasia and heterogeneity. Therefore, even if the tumour specimens are sampled from the primary site, there is still low cancer cell abundance in the specimens, and some KRAS mutations may be missed.

In summary, KRAS mutation subtypes have different distributions in different populations and can be used as biological indicators to predict the survival of patients after PDAC surgery.

## Data availability statement

The raw data supporting the conclusions of this article will be made available by the authors, without undue reservation.

## Ethics statement

The studies involving human participants were reviewed and approved by Ethics Committee of Hunan Provincial People’s Hospital. The patients/participants provided their written informed consent to participate in this study.

## Author contributions

MD is mainly responsible for data analysis, paper writing, revision, and translation. RJ was mainly responsible for the translation and polishing of the paper, as well as the writing of the paper. YL was mainly responsible for the data collection and processing of the paper. FY is mainly responsible for the data collection of the paper. JL is mainly responsible for data processing and ethics of the paper. XT was mainly responsible for the data collection and processing of the paper. KC was mainly responsible for the design of the paper, data collection, and help in the later revision of the paper. WC was mainly responsible for paper revision, data processing, clinicopathology and sequencing data acquisition. All authors contributed to the article and approved the submitted version.
